# And‐1 O‐GlcNAcylation regulates homologous recombination repair and radioresistance in colorectal cancer

**DOI:** 10.1002/ctm2.785

**Published:** 2022-04-26

**Authors:** Yuan Zhou, Yi Zhang, Changmin Peng, Zhuqing Li, Huadong Pei, Haiping Pei, Wenge Zhu

**Affiliations:** ^1^ Department of General Surgery Xiangya Hospital Central South University Changsha China; ^2^ Department of Biochemistry and Molecular Medicine The George Washington University School of Medicine and Health Sciences Washington USA; ^3^ GW Cancer Center The George Washington University Washington USA

Dear editor,

And‐1 is an important factor for end resection of homologous recombination (HR) repair, and elevated HR repair activity is one of the major mechanisms contributing to radioresistance in colorectal cancer.^1^ How And‐1 is regulated during HR repair and how HR repair is upregulated in radio‐resistant colorectal cancer remain largely unknown. This study elucidates a novel And‐1 O‐GlcNAcylation‐mediated mechanism regulating HR repair and radioresistance in colorectal cancer.

O‐GlcNAc glycosylation (O‐GlcNAcylation) is a dynamic posttranslational modification through which a single O‐linked β‐N‐acetylglucosamine (O‐GlcNAc) is attached to serine and/or threonine residues by the enzyme O‐GlcNAc transferase (OGT) or reversibly removed by O‐GlcNAcase (OGA).^2^, ^3^ Although O‐GlcNAcylation has been shown to involve in HR repair,^4^, ^5^ the detailed mechanism by which O‐GlcNAcylation regulates HR repair is still poorly understood. To explore the role of OGT in HR repair, we measured repair activity using a stable cell line with two nonfunctional green fluorescent protein (GFP) alleles that are activated after HR repair.^6^ In cells treated with siOGT or an OGT inhibitor (OSMI‐1), the efficiency of HR repair decreased significantly (Figures 1A, S1A), consistent with previous observations.^4^, ^5^ OGT inhibition in HT‐29 and SW620 cells significantly reduced cell survival in response to ionizing radiation (IR) (Figure 1B). Strikingly, And‐1 interacted with OGT in vivo and in vitro (Figures 1C,D, S1B), and IR or etoposide increased the interaction of And‐1 with OGT (Figure 1E,F). An in vitro O‐GlcNAcylation assay showed that His‐OGT (KD) directly O‐GlcNAcylated GST‐And‐1 (Figure S1C), and coexpression of His‐OGT(KD) with GST‐And‐1 in *Escherichia coli* led to And‐1 O‐GlcNAcylation (Figure 1G). Moreover, GST‐OGA decreased the level of O‐GlcNAcylated And‐1 in vitro (Figure 1H), and IR significantly increased And‐1 O‐GlcNAcylation (Figure 1I). Thus, And‐1 is a direct target of OGT.

**FIGURE 1:**
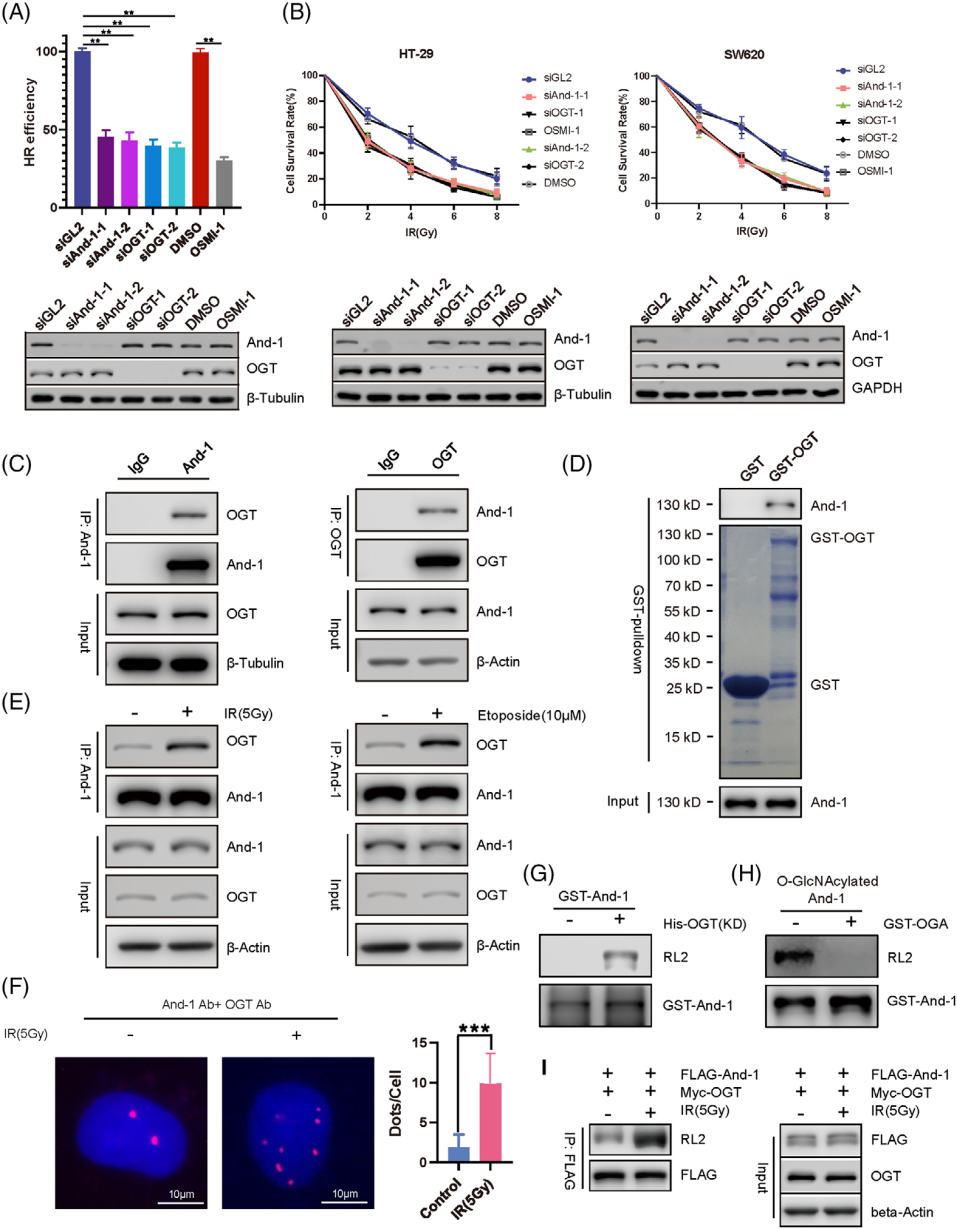
And‐1 interacts with O‐GlcNAc transferase (OGT) and is O‐GlcNAcylated. (A) O‐GlcNAcylation is required for homologous recombination (HR) repair. Direct repeat green fluorescent protein (DR‐GFP) HR reporter cells were transfected with control siGL2, siAnd‐1, siOGT or OGT inhibitor (OSMI‐1), followed by transfection with I‐SceI for 48 hrs. The resulting cells were then subjected to flow cytometric analysis for GFP‐positive cells. Upper panel, quantification of data from the HR report assay. Error bars correspond to the means ± SEM (*n* = 3; *, *p* ≤ .05, two‐tailed Student's *t* test). Lower panel, western blotting for And‐1 and OGT in cells treated in the upper panel. (B) Survival of the indicated colorectal cancer cells that were transfected with the indicated siRNAs or treated with OSMI‐1, followed by ionizing radiation (IR). Lower panel, western blotting for the indicated proteins in cells shown in the upper panel. Data represent the means ± SD from three independent experiments. *, *p* ≤ .05. (C) Coimmunoprecipitation (co‐IP) assay to detect the interactions of And‐1 with OGT in 293T cells. (D) Co‐IP assay to detect the interaction of And‐1 with OGT in vitro. Co‐IP assays were performed by using recombinant GST‐OGT and His‐And‐1 proteins purified from *Escherichia coli*. (E) Co‐IP assay to detect the interactions of And‐1 with OGT before and after ionizing radiation or camptothecin (CPT) treatment in 293T cells. (F) Proximity ligation assay (PLA) to examine the associations of And‐1 with OGT in U2OS cells. The red foci represent the PLA signals. Right panel, quantification of the mean fluorescence intensity (MFI) of PLA signals shown on the left. One hundred cells from each experiment were counted and analyzed. Data represent the means ± SD from three independent experiments. ***, *p* ≤ .001. (G) And‐1 is O‐GlcNAcylated by OGT in vitro. GST‐And‐1 and His‐OGT (kinetic domain) were cotransfected into BL21 *E. coli*, GST‐And‐1 was immunoprecipitated, and And‐1 O‐GlcNAcylation was detected by western blotting. (H) O‐GlcNAcylated And‐1 proteins were mixed with or without purified OGA, followed by examination of And‐1 O‐GlcNAcylation using western blotting. (I) Western blotting analysis of And‐1 O‐GlcNAcylation in 293T cells with or without IR treatment

O‐GlcNAcylation of And‐1 was observed as early as 5 minutes after IR treatment (Figure 2A), and accumulation of And‐1 and OGT at double‐strand breaks (DSBs) occurred approximately 3–5 minutes post‐DSB induction (Figures 2C, S2A). Consistent with our previous observations,^7^ the recruitment of And‐1 to chromatin was observed 5–10 minutes post‐IR treatment, which was before CtIP (Figure 2B). Thus, And‐1 O‐GlcNAcylation occured at the early step of HR repair (Figure S2B). O‐GlcNAcylation of And‐1 was only detected on its SepB domain (336‐984) (Figure 2D). Using both liquid chromatography‐mass spectrometry (LC‐MS) and an O‐GlcNAcylation prediction tool, we identified 18 potential O‐GlcNAcylation sites on SepB (Figure 2E,F, Tables S1,S2). Mutagenesis studies showed that mutations of S575A or S893A decreased And‐1 O‐GlcNAcylation (Figure S2C,D), and double mutations (S575A and S893A, named 2SA) significantly decreased O‐GlcNAcylation levels (Figure 2G). We next expressed wild‐type (WT) FLAG‐And‐1 or mutant FLAG‐And‐1(2SA) in SW620 cells with endogenous And‐1 depletion by shRNA (Figure S3A). Significantly, the recruitment of And‐1(2SA) but not WT And‐1 to DSB sites or chromatin was compromised (Figures 3A,B, S3C), suggesting a critical role of O‐GlcNAcylation in And‐1′s accumulation onto DSBs.

**FIGURE 2:**
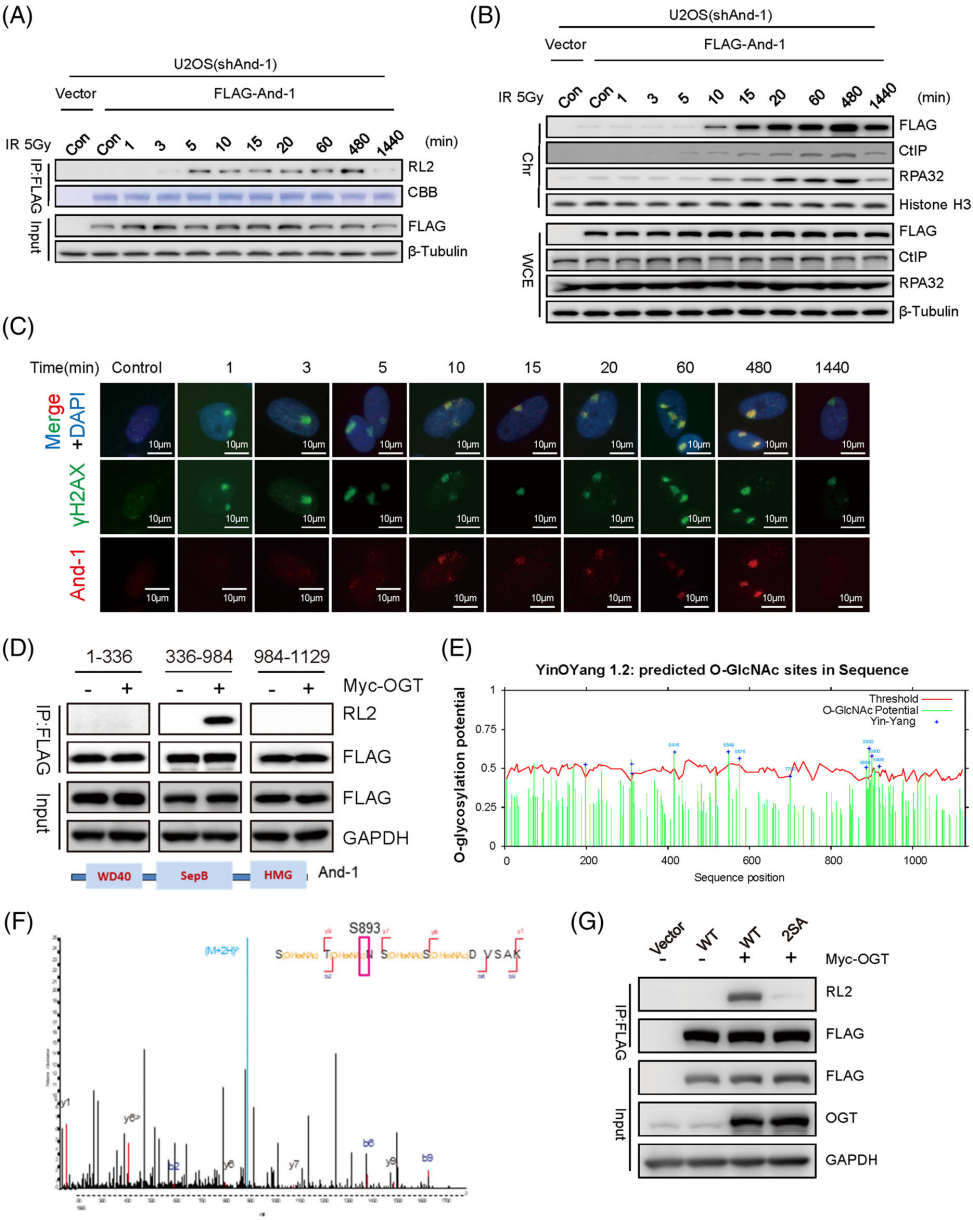
Dynamics of And‐1 O‐GlcNAcylation and identification of And‐1 O‐GlcNAcylation sites. (A) FLAG‐And‐1 was precipitated from cells at the indicated time points after ionizing radiation (IR) treatment. FLAG‐IPs were then resolved on SDS‐PAGE gel, followed by immunoblotting for the indicated proteins. (B) Cells transfected with vector or FLAG‐And‐1 were treated with or without IR. Cells were harvested at different time points after IR treatment, and chromatin fraction (Chr) and whole‐cell extracts (WCE) were resolved on SDS‐PAGE gel, followed by immunoblotting for the indicated proteins. (C) Immunofluorescence to examine the accumulation of And‐1 and γ‐H2AX on ultraviolet C (UVC)‐induced double‐strand break (DSB). (D) The indicated FLAG‐And‐1 truncated mutants were precipitated from 293T cells treated as indicated, followed by immunoblotting for the indicated proteins. (E) Predicted And‐1 O‐GlcNAcylation sites using the prediction program YinOYang 1.2. (F) Mass‐spec analyses of And‐1 O‐GlcNAcylation sites. The identified O‐GlcNAcylation site 893 is indicated. (G) Double mutations (S575A, S893A, named 2SA) reduced the levels of And‐1 O‐GlcNAcylation. FLAG‐And‐1 and mutants as indicated were precipitated from 293T cells, followed by immunoblotting for the indicated proteins

**FIGURE 3:**
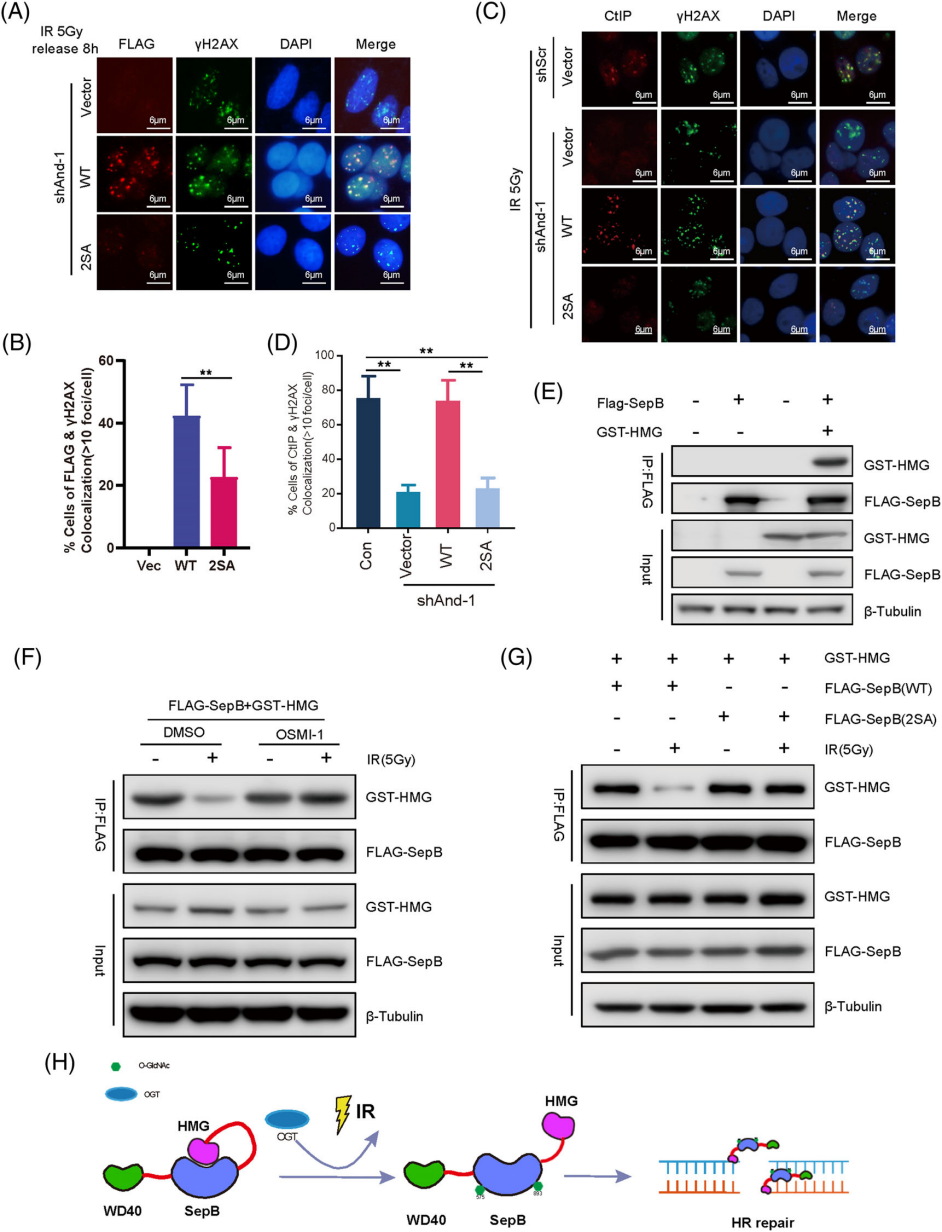
And‐1 O‐GlcNAcylation is required for the accumulation of And‐1 and CtIP at double‐strand break (DSB) sites by regulating their intramolecular interaction. (A) SW620 cells with endogenous And‐1 depleted by shAnd‐1 were transfected with vector, And‐1 or And‐1 mutant (2SA). Cells were then treated with ionizing radiation (IR). Eight hrs post‐IR treatment, cells were subjected to immunofluorescence for the indicated proteins. (B) Quantification of the data shown in A. Data represent the means ± SD from three independent experiments, and more than 100 cells were counted for each group. **, *p* ≤ .01. (C) And‐1 O‐GlcNAcylation is required for the recruitment of CtIP to double‐strand breaks (DSBs). Cells treated as in A were immunostained for the indicated proteins. (D) Quantification of the data shown in C. Data represent the means ± SD from three independent experiments, and more than 100 cells were counted for each group. **, *p* ≤.01. (E) FLAG‐SepB, glutathione S‐transferase‐high‐mobility group (GST‐HMG) alone or both were expressed in 293T cells. FLAG‐IPs were then immunoblotted for the indicated proteins. (F) 293T cells expressing both FLAG‐SepB and GST‐HMG were treated with or without OSMI‐1, followed by treatment with or without IR. Cells were then harvested, and FLAG‐IPs were immunoblotted for the indicated proteins. (G) 293T cells expressing GST‐HMG together with FLAG‐SepB or FLAG‐SepB (S2A) were treated with or without IR. Cells were then harvested 2 hrs post‐treatment, and FLAG‐IPs were immunoblotted for the indicated proteins. (H) Schematic of the mechanism of And‐1 O‐GlcNAcylation in the regulation of And‐1 structure and HR repair

We and others have shown that And‐1 promotes end resection by facilitating the recruitment of CtIP to DSBs.^7^, ^8^ Thus, it was not surprising to see that DSB site accumulation of CtIP was compromised in And‐1‐depleted cells with expression of And‐1(2SA) but not WT And‐1(Figure 3C,D). The accumulation of RPA32 on DSB sites was inhibited in And‐1‐depleted cells with expression of And‐1(2SA) (Figure S3D), indicating a defect in end resection. A similar observation was also seen in U2OS cells (Figures S3B, S3E‐H), suggesting that the role of And‐1 O‐GlcNAcylation in end resection is not limited to colorectal cancer cells.

And‐1 formed an intramolecular interaction between its high‐mobility group (HMG) and SepB domains (Figure 3E). We therefore assumed that And‐1 O‐GlcNAcylation may affect its structure. Indeed, the HMG‐SepB interaction was decreased in cells treated with IR, and the reduced interaction was restored upon treatment with OSMI‐1 (Figure 3F). The decreased HMG‐SepB interaction gradually recovered after IR treatment (Figure S3I). Significantly, the interaction of the mutant SepB (2SA) domain with HMG was not decreased in response to IR (Figure 3G). Thus, O‐GlcNAcylation of And‐1 prevents the intramolecular interaction between HMG and SepB, allowing the interaction of And‐1 with DNA at damage sites via its HMG domain (Figure 3H). To support the role of And‐1 O‐GlcNAcylation in end resection, the expression of WT but not mutant And‐1(2SA) restored the phosphorylation levels of Chk1 and RPA in And‐1‐depleted cells treated with IR or etoposide (Figures S4A,B, S4D), and the interaction between CtIP and NBS1 increased dramatically in cells expressing WT And‐1 but not And‐1(2SA) (Figure S4C).

To explore the role of And‐1 in radioresistance in colorectal cancer cells, we established two radio‐resistant colorectal cancer cell lines (named HT‐29 RR and SW620 RR) (Figure S5A‐C). After IR treatment, the reduction in γ‐H2AX levels in the two resistant cell lines was much faster than that in the sensitive cell line (Figures S5D,E), indicating elevated repair activity in resistant cells. Strikingly, OGT and And‐1 O‐GlcNAcylation expression levels were increased in two resistant cells compared to their sensitive counterparts (Figure 4A,B). Resistant cells became sensitive to IR when And‐1 was depleted by shRNA (Figure 4C), suggesting a vital role of And‐1 in the regulation of radioresistance. In resistant SW620 RR cells with downregulation of endogenous And‐1 by shRNA, expression of WT And‐1 but not And‐1(2SA) quickly reduced the levels of γH2AX or DSBs post‐IR treatment (Figure 4D,E), indicating a key role of And‐1 O‐GlcNAcylation in HR repair of resistant cells. Cells expressing WT And‐1 exhibited a higher survival rate and resistance to apoptosis than cells expressing And‐1(2SA) (Figures 4F,G, S5F). The increased cell survival in cells expressing WT And‐1 appeared to be dependent on And‐1 O‐GlcNAcylation because OGT depletion restored the sensitivity of these cells to IR (Figure 4H). We recently identified two potent And‐1 inhibitors, CH3 and bazedoxifene (BZA).^9^ Inhibition of And‐1 by these two inhibitors resensitized the resistant cells to IR (Figures 4I,J, S5G).

**FIGURE 4:**
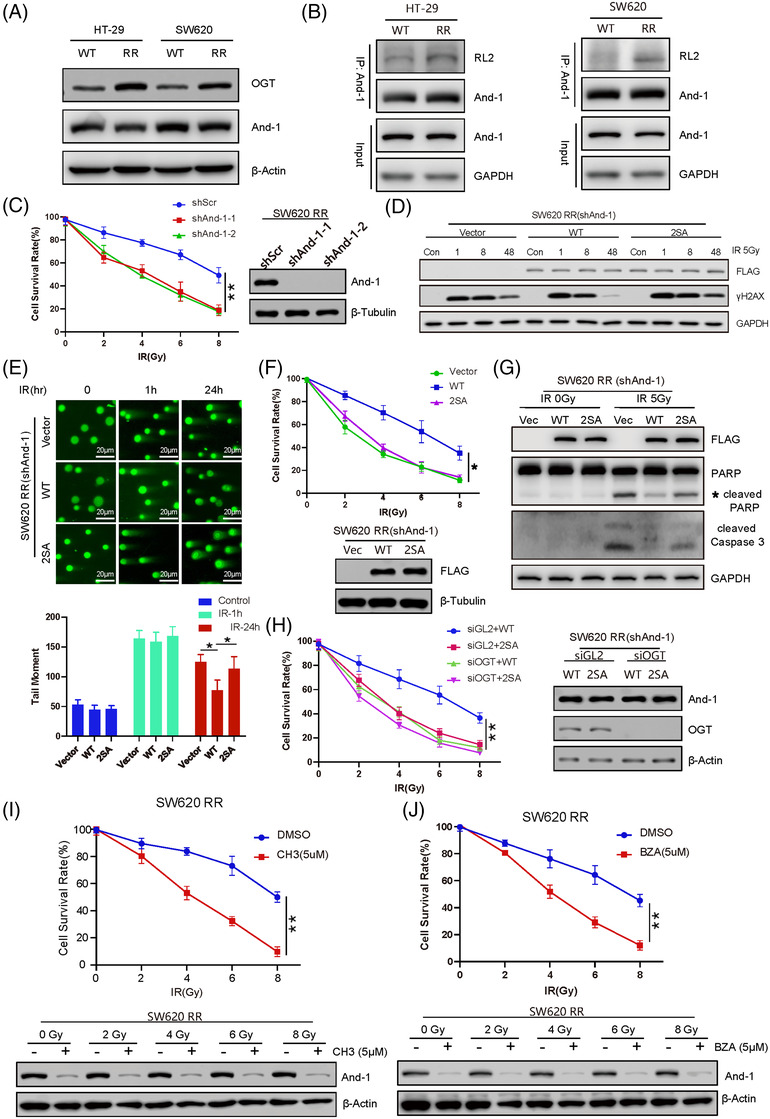
Increased And‐1 O‐GlcNAcylation contributes to radioresistance in colorectal cancer cells. (A) O‐GlcNAc transferase (OGT) and And‐1 protein expression in the indicated ionizing radiation (IR)‐sensitive and IR‐resistant cells. The indicated protein expression levels were examined by western blotting. (B) And‐1 O‐GlcNAcylation levels in the indicated IR‐sensitive and IR‐resistant cells. And‐1 was precipitated from the indicated cells, and immunoprecipitates (IPs) were then immunoblotted for the indicated proteins. (C) SW620 RR cells with And‐1 downregulation by shAnd‐1 (shAnd‐1‐1, shAnd‐1‐2) were treated with IR at the indicated doses. The survival of cells was determined by an SRB assay 72 hrs after treatment. Right panel, western blotting of the indicated proteins from cells treated in the left panel. (D) SW620 RR cells with downregulated endogenous And‐1 by shAnd‐1 cells were transfected with And‐1 and And‐1 mutant (2SA), followed by treatment with IR. Cells were then harvested at the indicated time points for western blotting to detect the expression of the indicated proteins. (E) SW620 RR cells with depleted endogenous And‐1 by shRNA were transfected with empty vector (Vector), wild‐type (WT) And‐1 or mutant And‐1(2SA), followed by irradiation with 5 Gy. Cells were then subjected to the comet assay at the indicated time points. Right panel, quantification of the tail moment in each treatment. Data represent the means ± SD from three independent experiments. (F) SW620 RR cells with endogenous And‐1 depletion by shRNA were transfected with empty vector (Vector), WT And‐1 or mutant And‐1(2SA). Cells were then irradiated with the indicated doses of IR. The survival of cells was measured by SRB assay 72 hrs post‐IR treatment. Data represent the means ± SD from three independent experiments. Lower panel, western blotting to detect the expression of the indicated proteins. (G) SW620 RR cells with endogenous And‐1 depleted by shRNA were transfected with empty vector (Vector), WT And‐1 or mutant And‐1(2SA). Cells were then irradiated with the indicated doses of IR. Forty‐eight hrs after IR, the cells were lysed and the expression levels of the indicated proteins were examined by western blotting. (H) SW620 RR cells with endogenous And‐1 depletion by shRNA were transfected with WT And‐1 or mutant And‐1(2SA) and then treated with siGL2 or siOGT. Cells were then irradiated with the indicated doses of IR. Left panel, the survival of cells was measured by SRB assay 72 hrs post‐IR treatment. Data represent the means ± SD from three independent experiments. Right panel, western blotting to detect the expression of the indicated proteins in the cells shown in the left panel. (I) SW620 RR cells were treated with DMSO or CH3 together with the indicated doses of IR. Left panel, the survival of cells was measured by SRB assay 72 hrs post‐IR treatment. Data represent the means ± SD from three independent experiments. **, *p* ≤ .05. Lower panel, western blotting indicated proteins in cells treated with the indicated doses of CH3. (J) SW620 RR cells were treated with dimethyl sulfoxide (DMSO) or bazedoxifene (BZA) together with the indicated doses of IR. Upper panel, the survival of cells was measured by SRB assay 72 hrs post‐IR treatment. Data represent the means ± SD from three independent experiments. **, *p* ≤ .05. Lower panel, western blotting indicated proteins in cells treated with the indicated doses of BZA

In conclusion, our study elucidates a novel And‐1 O‐GlcNAcylation‐mediated mechanism regulating HR repair and radioresistance in colorectal cancer. Since BZA is an FDA‐approved drug for the treatment of symptoms associated with menopause,^10^ our study reveals a potential clinical trial for radio‐resistant colorectal cancer patients in the near future.

## CONFLICT OF INTERESTS

The authors declare no conflict of interests.

## Supporting information



SUPPORTING INFORMATION
