# Supplementary material for: And‐1 O‐GlcNAcylation regulates homologous recombination repair and radioresistance in colorectal cancer
Source: Clin Transl Med. 2022 Apr 26;12(4):e785. doi: 10.1002/ctm2.785 (PMC9043118; doi:10.1002/ctm2.785)
Supplement: Supplementary file 1 — SUPPORTING INFORMATION [file CTM2-12-e785-s001.pdf]

Supplemental information

And-1 O-GlcNAcylation regulates HR repair and radioresistance in colorectal cancer

Yuan Zhou, Yi Zhang, Changmin Peng, Zhuqing Li, Huadong Pei, Haiping Pei, Wenge Zhu

This supplemnetal inforamtion includes

1. Supplemtnal Figures S1-5
2. Supplemtnal Tables S1-2

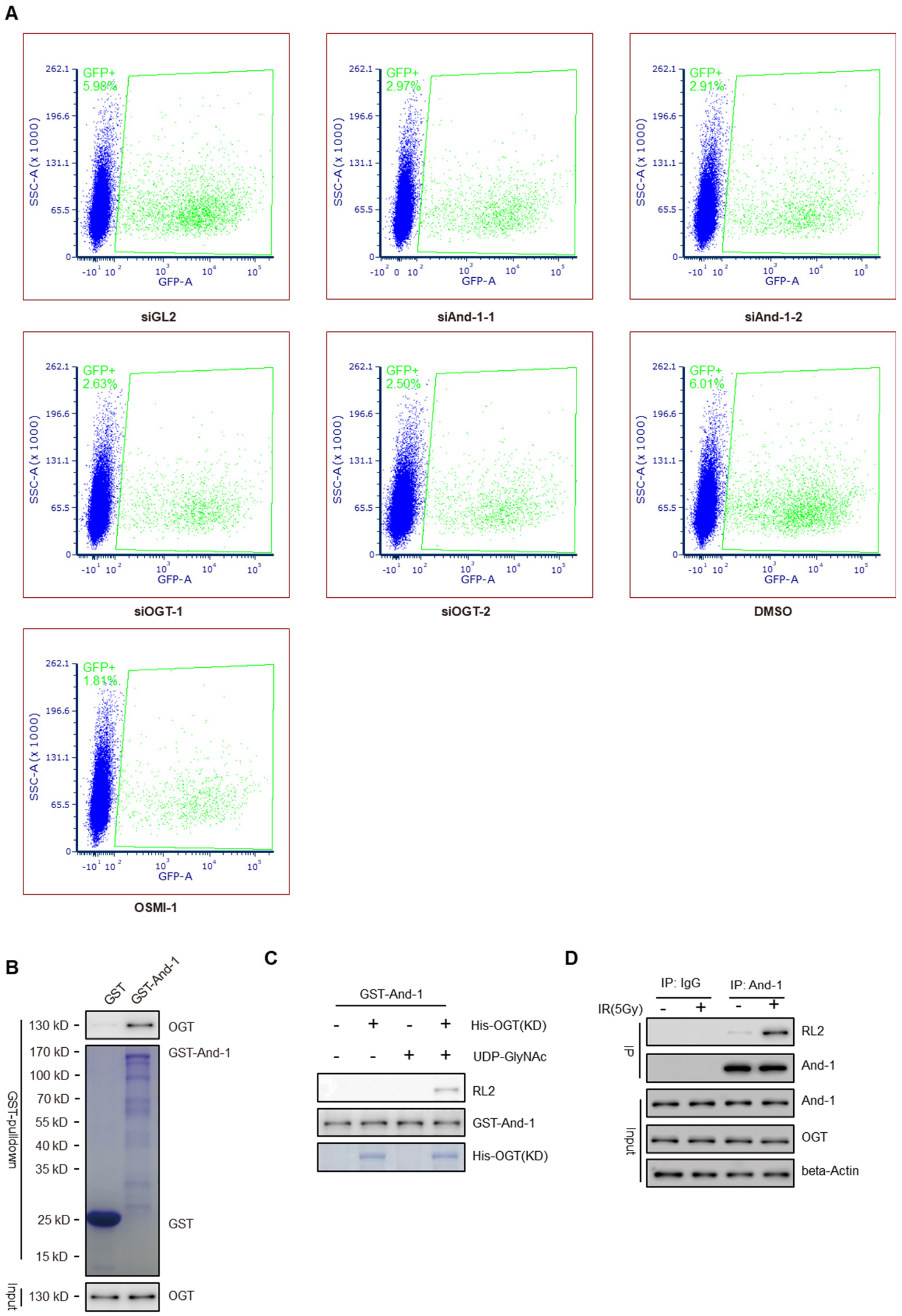

Supplemental Figure S1

(A) Representative Flow cytometry plots for Figure 1A. (B) Co-IP assay to detect the interaction of And-1 with OGT *in vitro*. Co-IP assays were performed by using recombinant GST-And-1 and His-OGT purified from E. coli. (C) OGT O-GlcNAcylates And-1 *in vitro*. *In vitro* glycosylation assay was performed by using GST-And-1 as the substrate. GST-And-1 was incubated with His-OGT for *in vitro* reaction. O-GlcNAcylated And-1 was blotted with RL2 antibody, and the total GST-And-1 protein was detected by Western blotting. Representative blots from three independent experiments were shown. (D) Western blotting analysis of endogenous And-1 O-GlcNAcylation from SW620 cells with or without IR treatment.

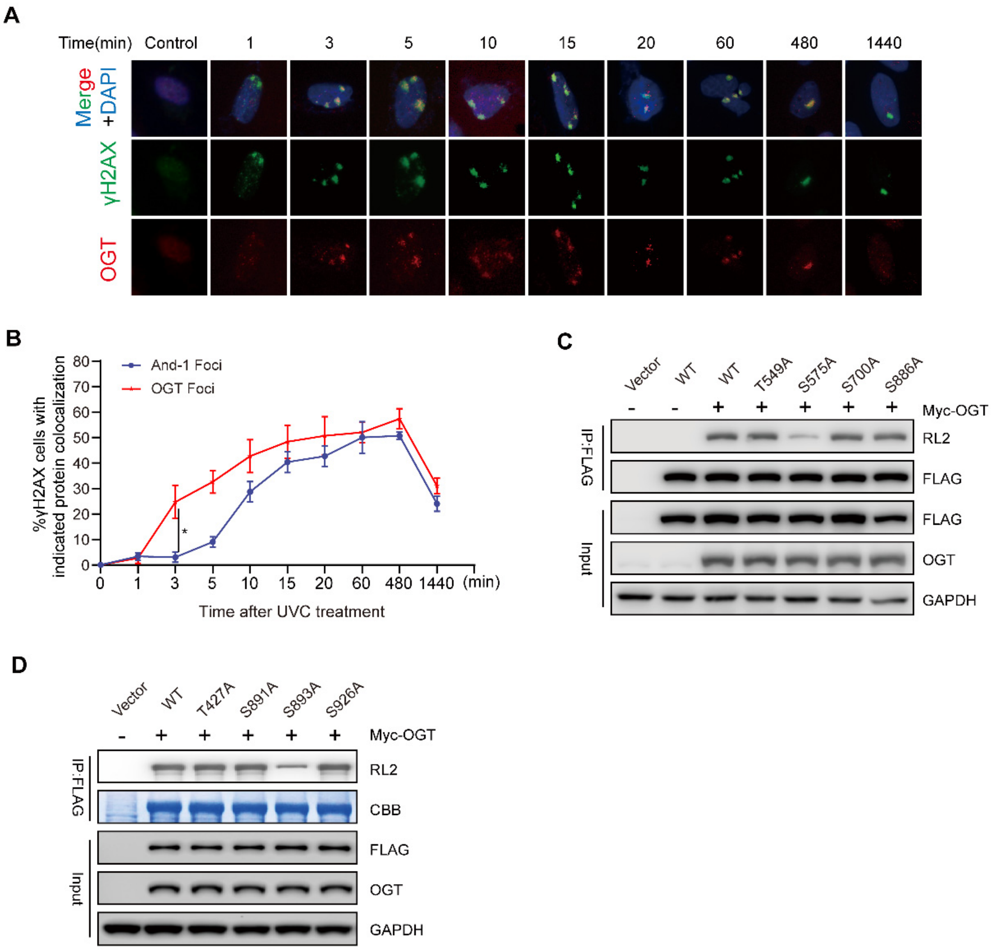

Supplemental Figure S2

(A) Immunofluorescence to examine the accumulations of OGT and γ-H2AX to UVC-induced DSB. (B) Quantification of results shown in Figure 2C and Figure S2A . Data represent means ± SD from three independent experiments. *$p \leq 0.05$. (C) FLAG-And-1 and mutants as indicated were precipitated from 293T cells, followed by immunoblotting for indicated proteins. (D) FLAG-And-1 and its mutants as indicated were precipitated from 293T cells, followed by immunoblotting for indicated proteins.

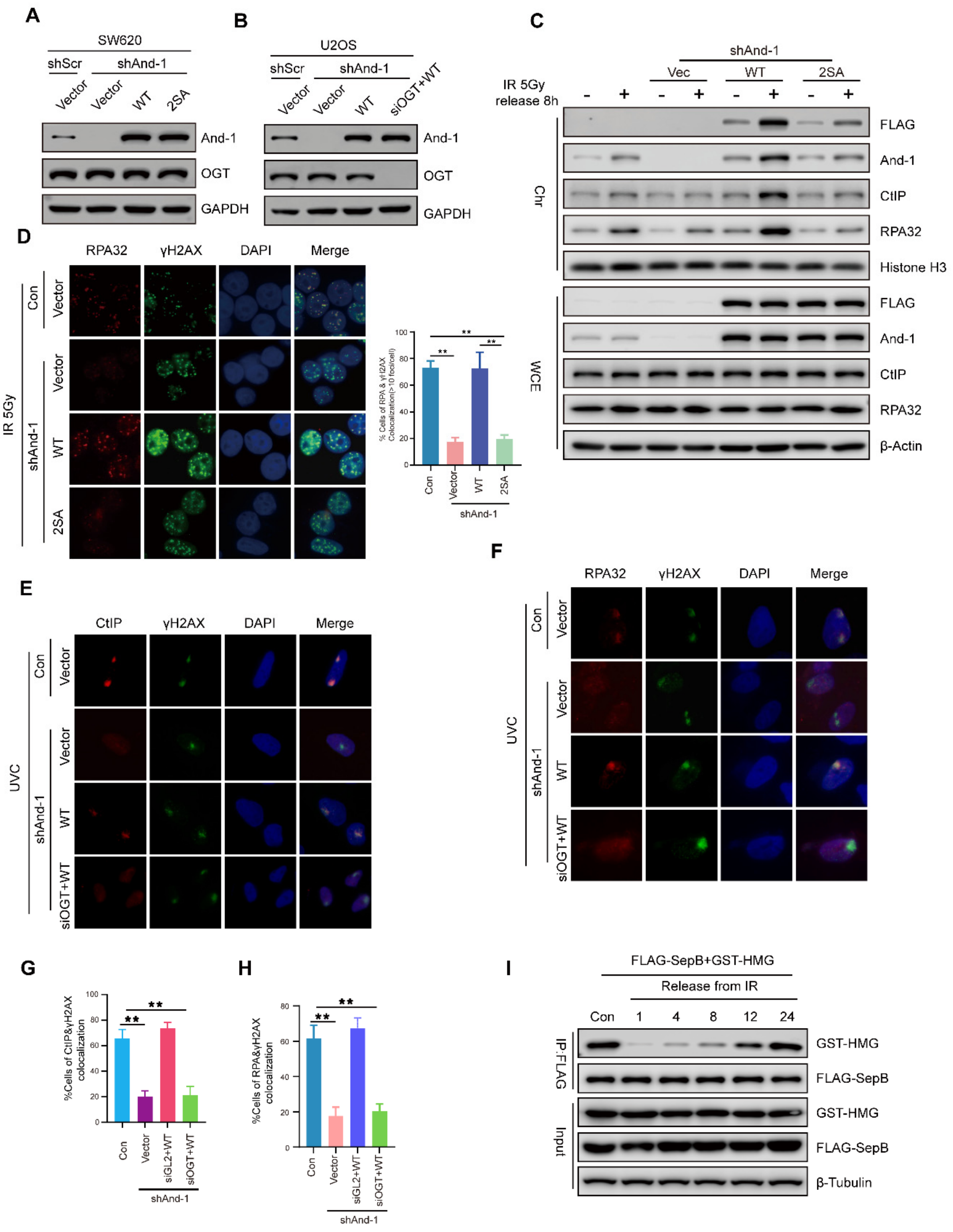

Supplemental Figure S3

(A) Western blotting for indicated proteins in cells used for experiments described in Figure 3B, Figure S3D; (B) Western blotting for indicated proteins in cells used for experiments described in Figure S3E-F. (C) SW620 cell with depleted endogenous And-1 by shAnd-1 were transfected with vector, And-1 or And-1 mutant (2SA). Cells were then treated with or without IR. 8 hours post IR treatment, cells were harvested. The chromatin fraction (Chr) and whole-cell extraction (WCE) were extracted from harvested cells and immunoblotted for indicated proteins. (D) And-1 O-GlcNAcylation is required for the recruitment of RPA32 to

DSBs. Cells treated as in C were immunostained for indicated proteins. Right panel, quantification of RPA32 signal. Data represent means ± SD from three independent experiments, more than 100 cells were counted for each group. **, $p \leq 0.01$. (E) And-1 O-GlcNAcylation is required for the recruitment of CtIP to DSBs. U2OS or U2OS cell with depleted endogenous And-1 by shAnd-1 were transfected with vector, And-1, or And-1 with OGT knockdown by siRNA. DSBs were induced in these cells by UVC. 8 hours post UVC treatment, IF assays were performed for indicated proteins. (F) And-1 O-Glycosylation is required for the recruitment of RPA32 to DSBs. U2OS or U2OS cell treated as in E. IF assay was performed for indicated proteins. (G) Quantification of CtIP foci intensity shown in E. Data represent means ± SD from three independent experiments, more than 100 cells were counted for each group. **, $p \leq 0.01$. (H) Quantification of RPA32 foci intensity shown in F. Data represent means ± SD from three independent experiments, more than 100 cells were counted for each group. **, $p \leq 0.01$. (I) 293T cells with expression of both of FLAG-SepB and GST-HMG were treated with IR. Cells were then harvested at indicated time points post treatment. FLAG-IPs were immunoblotted for the indicated proteins.

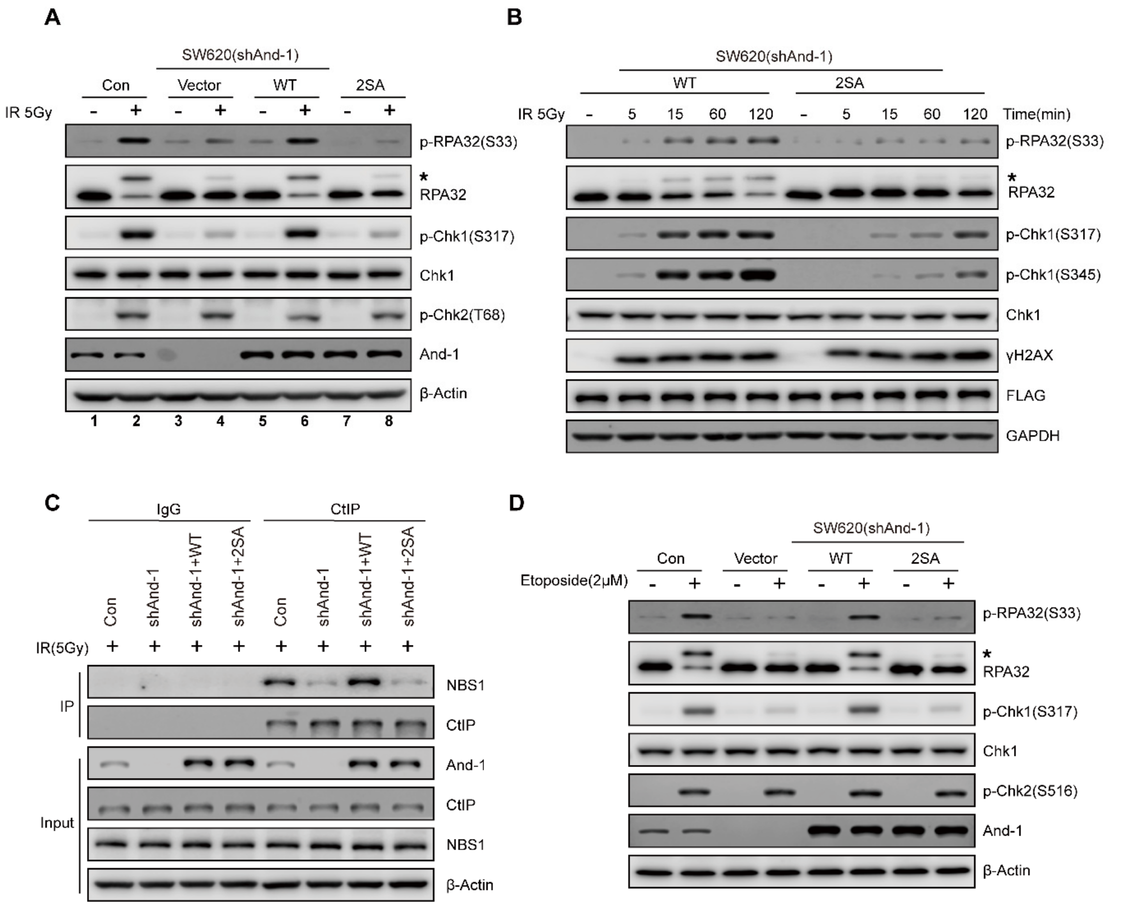

Supplemental Figure S4

(A) SW620 or SW620 cell with depleted endogenous And-1 by shAnd-1 were transfected with vector, And-1 or And-1 mutant (S2A). Cells were then treated with or without IR. 2 hours post IR treatment, cells were harvested for Western blotting for indicated proteins. (B) SW620 cells with depleted endogenous And-1 by shAnd-1 were transfected with And-1 or And-1 mutant (2SA), and then treated with IR. Harvested at indicated time points post treatment were immunoblotted for indicated proteins. (C) SW620 or SW620 cells with endogenous And-1 depletion by shRNA were transfected with indicated plasmids. 2 hours after treatment with IR, cells were harvested for IP of CtIP or IgG, followed by immunoblotting for indicated proteins. (D) SW620 or SW620 cells with endogenous And-1 depletion by shRNA were transfected with indicated plasmids. Cells were then treated with etoposide. 2 hours after treatment, cells were harvested and immunoblotted for indicated proteins. Asterisks in A, B and D: hyper phosphorylated RPA32.

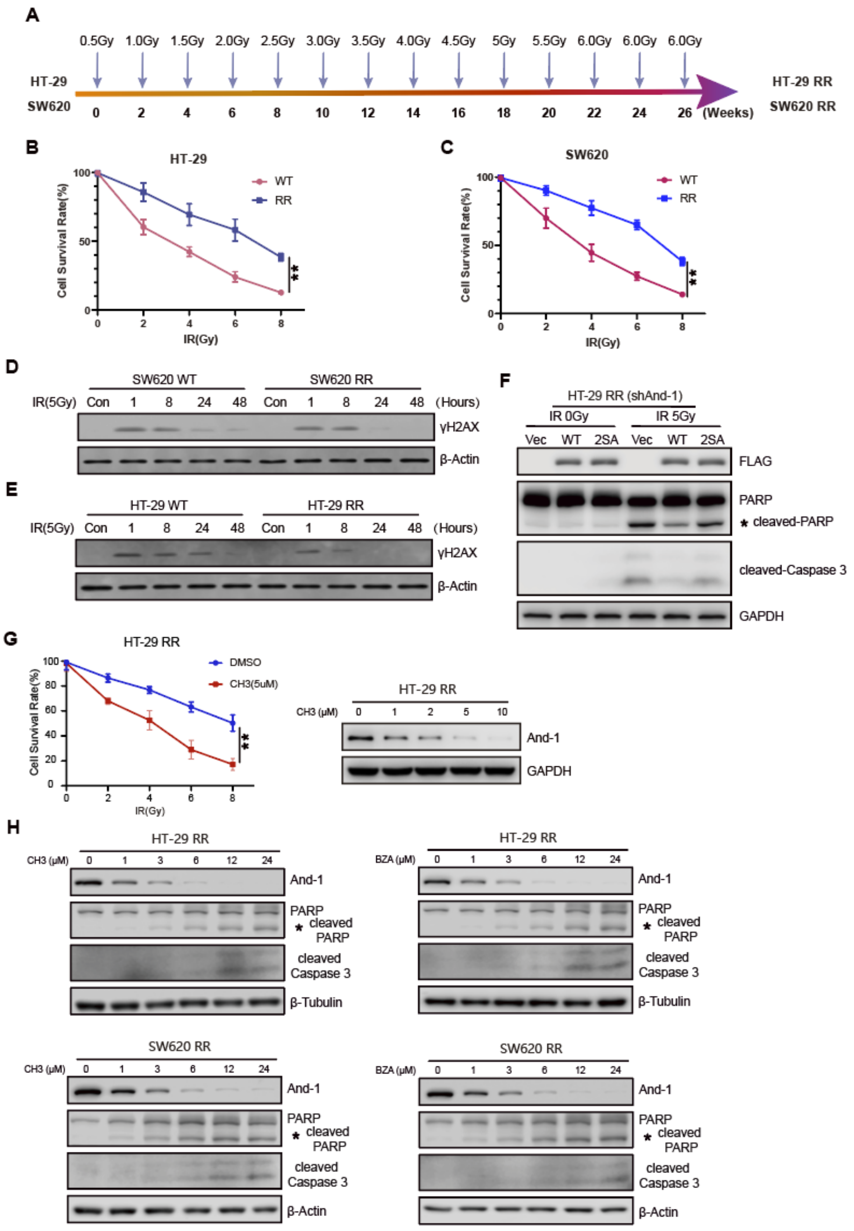

Supplemental Figure S5

(A) Schematic diagram depicts the procedure to generate radioresistant HT-29 RR and SW620 RR cells. (B-C) Survival of HT-29 and SW620 sensitive and resistant cell lines treated with indicated doses of IR. The survival of cells was measured by SRB assay after 72 hours post IR treatment. Data represent means ±SD from three independent experiments. **, $p \leq 0.05$. (D-E) Western blotting for indicated proteins in paired sensitive and radioresistant cells after 5 Gy radiation treatment, followed by harvesting at different time point. (F) HT-29 RR cells with depleted And-1 by shRNA were transfected with empty vector (Vector), WT And-1 or mutant And-1(2SA). Cells were then irradiated with indicated doses of IR. 48 hours after IR, cells were lysed and the expression levels of indicated proteins were examined by Western blotting. (G) Radioresistant HT-29 RR cells were treated with And-1 inhibitor CH3 and indicated doses of IR. The survival of cells was measured by SRB assay 72 hours post IR

treatment. Data represent means ±SD from three independent experiments. **, $p \leq 0.05$. Right panel, Western blotting for indicated proteins in cells shown in left panel.

(H) Radioresistant HT-29 RR cells and SW620 RR cells were treated with And-1 inhibitor CH3 or BZA at different concentration. Cells were harvested at 72 hours after treatment. Indicated proteins were detected by western blotting.

Supplemental Table S1: Predicted And-1 O-GlcNAcylation sites on SepB domain by YinOYang 1.2 (http://www.cbs.dtu.dk/services/YinOYang/).

SeqName	Residue	O-GlcNAc result	Potential(O-GlcNAc)	Thresh.(1)	Thresh.(2)	NetPhos potential (Thresh=0.5)	YinOYang?
Sequence	415 S	+	0.6122	0.4995	0.6237		
Sequence	549 S	+	0.5992	0.5226	0.6548		
Sequence	575 S	+	0.5738	0.5238	0.6564		
Sequence	700 T	+	0.4597	0.4534	0.5615		
Sequence	886 S*	+	0.5066	0.4420	0.5461	0.527	*
Sequence	893 S*	++	0.6280	0.4459	0.5514	0.735	*
Sequence	900 S*	++	0.5789	0.4650	0.5772	0.944	*
Sequence	906 S	+	0.4930	0.4917	0.6132		

Supplemental Table S2: LC-Mass-spec analyses of And-1 O-GlcNAcylation sites on SepB domain.

SeqName	Residue
Sequence	**427 T**
Sequence	**891 T**
Sequence	**893 S**
Sequence	**894 S**
Sequence	**900 S**
Sequence	**904 T**
Sequence	**906 S**
Sequence	**926 S**
Sequence	**929 S**
